# Effects of microbiota-driven therapy on inflammatory responses in elderly individuals: A systematic review and meta-analysis

**DOI:** 10.1371/journal.pone.0211233

**Published:** 2019-02-06

**Authors:** Hua Qu, Ying Zhang, Hua Chai, Zhu-ye Gao, Da-zhuo Shi

**Affiliations:** 1 Xiyuan Hospital, China Academy of Traditional Chinese, Beijing, China; 2 China Heart Institute of Chinese Medicine, China Academy of Chinese Medical Sciences, Beijing, China; University of Mississippi Medical Center, UNITED STATES

## Abstract

Current evidence suggests that age-associated inflammation, a strong risk factor for the health status of elderly individuals, is closely associated with gut microbiota. Previous animal studies have demonstrated a benefit of microbiota-driven therapy in decreasing low-grade chronic inflammation in elderly individuals; however, it remains controversial in clinical studies. Therefore, the present systematic review and meta-analysis were designed to assess the effects of microbiota-driven therapy on inflammatory markers in elderly individuals. PubMed, EMBASE, and the Cochrane Library were searched with no language restrictions from the inception of the database to November 11th, 2018 to identify all existing literature. We calculated pooled standard mean difference (SMD) using fixed effect model or random effect model to assess the effects of microbiota-driven therapy on elderly individuals. The methodological quality of the studies was determined according to the Cochrane Handbook. The publication bias was evaluated by funnel plot and Egger regression test. Ten randomized controlled studies, with 689 elderly individuals (347 individuals in the microbiota-driven therapy group and 342 individuals in the placebo group), were included in the analysis. Compared with placebo, microbiota-driven therapy did not decrease the levels of tumor necrosis factor-α (SMD, -0.24; 95% CI, -0.69 to 0.21; p = 0.30; I^2^ = 82.7%), interleukin-6 (SMD, -0.13; 95% CI, -0.74 to 0.49; p = 0.69; I^2^ = 90.7%) and interleukin-10 (SMD, 1.00; 95% CI, -0.15 to 2.15; p = 0.09; I^2^ = 96.3%). In addition, the microbiota-driven therapy also did not decrease the levels of C reactive protein (SMD, -1.28; 95% CI, -2.62 to 0.06; p = 0.06; I^2^ = 96.2%), interleukin-1β (SMD, -0.22; 95% CI, -0.81 to 0.37; p = 0.46; I^2^ = 73.8%), interleukin-8 (SMD, -0.03; 95% CI, -0.67 to 0.61; p = 0.93; I^2^ = 88.0%) and monocyte chemoattractant protein-1 (SMD, -0.11; 95% CI, -0.41 to 0.20; p = 0.49; I^2^ = 0%) when compared with placebo. No obvious publication bias was observed (p>0.05). In conclusion, the present meta-analysis of available randomized controlled studies did not suggest any significant benefit of microbiota-driven therapy in decreasing the inflammatory responses of elderly individuals.

## Introduction

Age-associated inflammation is a strong risk factor for the health status of elderly individuals. Previous studies have demonstrated that elderly individuals with higher levels of inflammatory markers are less independent and more likely to develop a variety of late-life diseases[1–4], accompanied by a higher hospitalization rate and all-cause mortality rate[5,6]. Age-associated inflammation has also been shown to increase susceptibility to pneumococcal infection[7,8] and has been associated with increased disease severity and decreased survival from coronary heart disease in elderly individuals[9,10].

The evidence has proven that some gut microbiota promote aging-associated inflammation and that reversing these microbiota changes represents a potential therapeutic effect on reducing age-associated inflammation. Microbiota-driven therapy, mainly including the intake of probiotics, prebiotics or symbiotics, seems a promising approach to manage age-associated inflammation. Previous animal studies have demonstrated that microbiota-driven therapy changed the composition of the gut microbiota and decreased inflammatory markers[11]; however, it remains controversial in clinical studies. Some studies have indicated that microbiota-driven therapy decreased inflammatory biomarkers, such as tumor necrosis factor α (TNF-α) and C-reactive protein (CRP)[12,13]; in contrast, other studies have suggested no beneficial effects of the therapy[14,15]. Therefore, the present meta-analysis of randomized controlled trials (RCTs) was designed to assess the effect of microbiota-driven therapy on the inflammatory responses in elderly individuals.

## Methods

This study was performed according to the guidelines of the 2009 Preferred Reporting Items for Systematic Reviews and Meta-Analysis statement (PRISMA)[16] (S1 Table). PRISMA is an evidence-based minimum set of items for reporting in systematic reviews and meta-analyses, which focuses on the reporting of reviews evaluating randomized trials[16]. The methodological quality of eligible studies was determined according to the recommendation of the Cochrane Handbook[17]. The Cochrane Handbook for systematic reviews of interventions contains methodological guidance for the preparation and maintenance of intervention reviews[17].

### Data source and search strategies

Two reviewers (Hua Qu and Ying Zhang) searched PubMed, EMBASE, and the Cochrane Library with no language restrictions from the inception of the database to November 2018 to identify all existing literature. The searching strategies are supplied in the S2 Table. A manual search was also performed to identify relevant references from the selected articles and published reviews. The studies were eligible if they met the following inclusion criteria: (1) the study described a randomized, controlled, parallel or crossover trial; (2) the participants were healthy elderly individuals with age>60 years; (3) the intervention group received microbiota-driven therapy (probiotic, prebiotic or symbiotic), and the compared group received placebo.

### Data extraction and assessment of study quality

Two reviewers (Hua Qu and Hua Chai) extracted data independently. If a disagreement occurred between them, it was resolved by consulting with the third investigator (Da-zhuo Shi). We would contact with authors if the article was only published in abstract form, and the studies with which we failed to obtain original data were excluded. The data extracted from the eligible studies were as follows: (1) the first author’s name and publication year, (2) intervention duration, (3) inclusion criteria, (4) intervention method, (5) number of individuals, (6) age of individuals, (7) percentage of males, and (8) clinical outcomes.

### Statistical analysis

In this meta-analysis, the outcomes, which are continuous data, are used to calculate standard mean difference (SMD) presenting with effect size and 95% confidence intervals (CI); and P<0.05 (two-sided hypothesis testing) indicating a statistically difference between microbiota-driven therapy group and placebo group. Interstudy variations and heterogeneities were estimated using Cochran’s Q-test, with Ph<0.05(two-sided hypothesis testing) indicating a statistically significant heterogeneity[18]. Furthermore, the effects of heterogeneity were quantified using the I^2^ test (range, 0–100%), which represented the proportion of interstudy variability that was able to be contributed to heterogeneity rather than to chance[19]. When a Q-test with Ph<0.05 or I^2^≥ 50%, the heterogeneity among studies was considered to be statistically significant and the random-effects model was chosen for the meta-analysis; otherwise, the fixed-effects model was used[20]. We performed a subgroup analysis to detect the potential sources of heterogeneity in the condition of I^2^≥ 50%[20]. In addition, the meta-regression provided a linear regression using a random effects model (I^2^≥50%) or a fixed effects model (I^2^<50%) and predicted effect size from a predictor variable[21]. Sensitivity analysis was performed to check the robustness of the pooled results by eliminating one study at a time. The publication bias was evaluated by a funnel plot and Egger’s regression test[22]. Statistical analysis was performed by using Stata (version 12.0). We have registered protocol for the present systematic review and meta-analysis, and the registered number of PROSPERO is CRD 42018116433.

## Results

### Description of included studies

**Fig 1:**
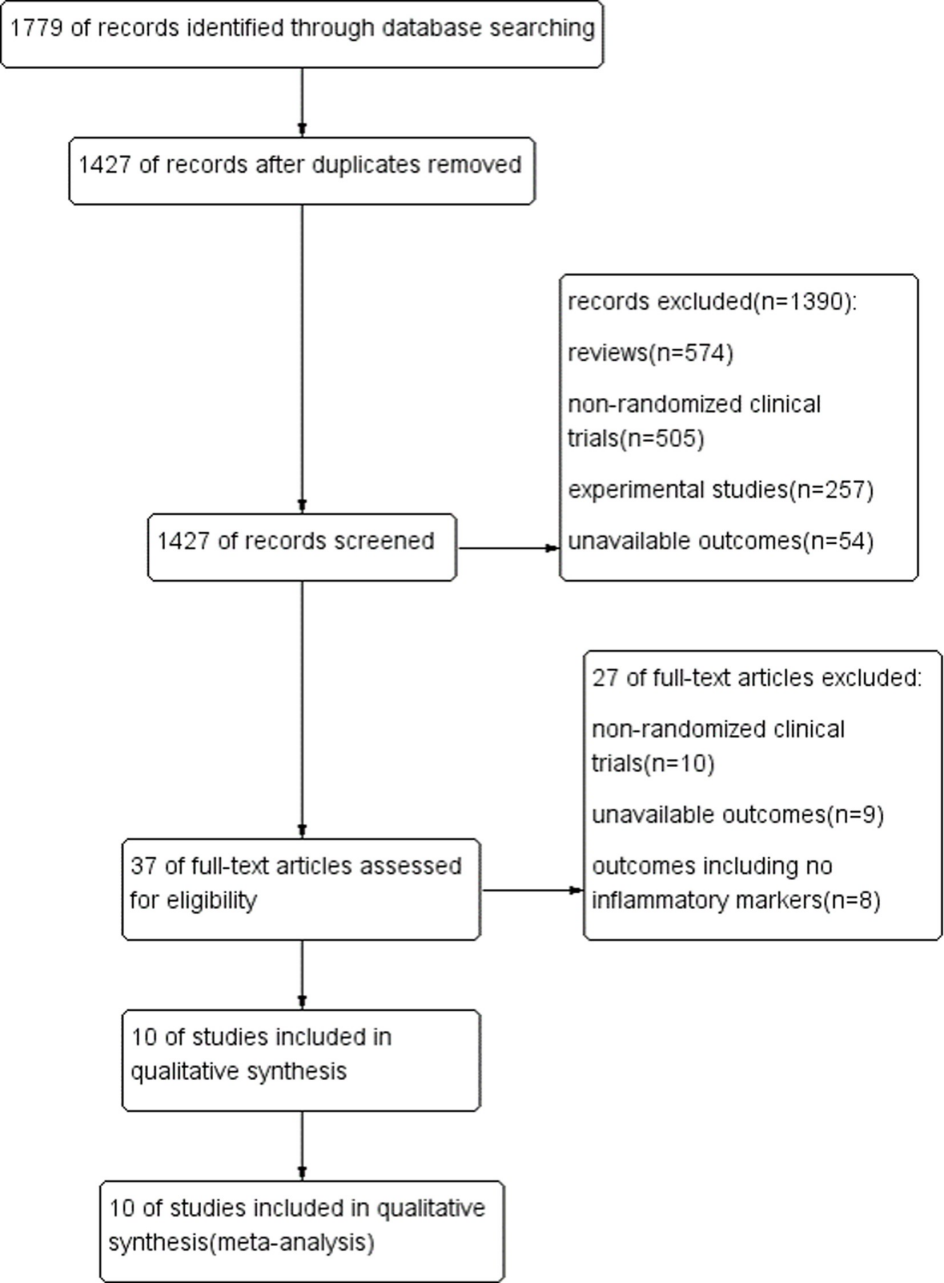
Literature search process and study selection.

One thousand seven hundred and seventy-nine studies (775 from PubMed, 655 from EMBASE and 349 from the Cochrane Library) were identified; 352 articles were excluded because of duplicated records. After the titles and abstracts of articles were screened, 1390 articles were excluded due the study being in review format, experimental studies or the study being an inappropriate design and/or unavailable outcomes. After the remaining 37 full-text articles were reviewed, 27 articles were excluded due to no-randomized clinical trials, unavailable outcomes or irrelevant outcomes. Finally, 10 studies[12–15,23–28] (5 randomized controlled crossover studies and 5 randomized controlled paralleled studies) published in English from 2008 to 2017, with sample sizes ranging from 36 to 88 individuals and intervention periods ranging from 14 days to 180 days, were entered into our meta-analysis (Fig 1, Table 1). The total number of elderly individuals was 689 (347 individuals in the microbiota-driven therapy group and 342 individuals in the placebo group). Overall, the effects of microbiota-driven therapy were evaluated based on TNF-α in 7 study arms (241 individuals in the microbiota-driven therapy group and 236 individuals in the placebo group)[12–15,23,26,27], interleukin-6 (IL-6) in 6 study arms (236 individuals in the microbiota-driven therapy group and 232 individuals in the placebo group)[12,13,15,23,24,26], interleukin-10 (IL-10) in 6 study arms (208 individuals in the microbiota-driven therapy group and 208 individuals in the placebo group)[12–15,26,28], CRP in 4 study arms (148 individuals in the microbiota-driven therapy group and 148 individuals in the placebo group)[13,24–26], interleukin-8 (IL-8) in 4 study arms (189 individuals in the microbiota-driven therapy group and 164 individuals in the placebo group) [12,13,15,24], interleukin-1β (IL-1β) in 2 study arms (87 individuals in the microbiota-driven therapy group and 87 individuals in the placebo group)[12,13] and monocyte chemoattractant protein-1 (MCP-1) in 2 study arms (84 individuals in the microbiota-driven therapy group and 80 individuals in placebo group)[13,23].

**Table 1:** Basic characteristics of individuals.

Study	Duration	Inclusion criteria	Intervention	Individuals (T/P)	Age(y)		Male(%)		Outcome
					T	P	T	P	
Park 2008	112 days	Age>60	prebiotic vs. placebo	78(41/37)	65.89 ±1.29	65.76 ±1.39	58.54	51.35	IL-6, TNF-α, MCP-1
Ouwehand 2008	180 days	age>60	probiotic vs. placebo	36(18/18)	84.3 ±0.98	84.3 ±0.98	N	N	IL-10, TNF-α
Costabile 2017	21 days	age 60–80	probiotic vs. placebo	74(37/37)	60–80	60–80	N	N	IL-6, IL-8, CRP
Scheid 2014	63 days	Age>60	prebiotic vs. placebo	74(37/37)	67.11±6.12	67.11±5.53	N	N	CRP
Valentini 2015	56 days	age 65–85	probiotic vs. placebo	62(31/31)	65–85	65–85	N	N	IL-6, IL-10, CRP, TNF-α
Ouwehand 2009	14 days	Age>65	probiotic vs. placebo	47(24/23)	70.3±7.2	71.7±6.2	20.8	30.4	TNF-α
Vulevic 2015	70 days	age 65–85	prebiotic vs. placebo	80(40/40)	65–85	65–85	N	N	IL-6, IL-8, IL-10, TNF-α
Vulevic 2008	70 days	age 64–79	prebiotic vs. placebo	88(44/44)	64–79	64–79	36.4	36.4	IL-1β, IL-6, IL-8, IL-10, TNF-α
Macfarlane 2013	28 days	age 65–90	synbiotic vs. placebo	86(43/43)	71.9±5.4	71.9±5.4	48.84	48.84	IL-1β, IL-6, IL-8, IL-10, TNF-α, CRP, MCP-1
Spaiser 2015	21 days	age 65–80	probiotic vs. placebo	44(22/22)	73.9±15.3	71.8±20	60.9	60.9	IL-10

Abbreviations: CRP, C reactive protein; TNF-α, tumor necrosis factor alpha; IL-1β, interleukin-1β; IL-6, interleukin-6; IL-8, interleukin-8; IL-10, interleukin-10; MCP-1, monocyte chemoattractant protein-1; T: microbiota-driven therapy group; P: placebo group; N, not clear.

### Quality assessment

**Fig 2:**
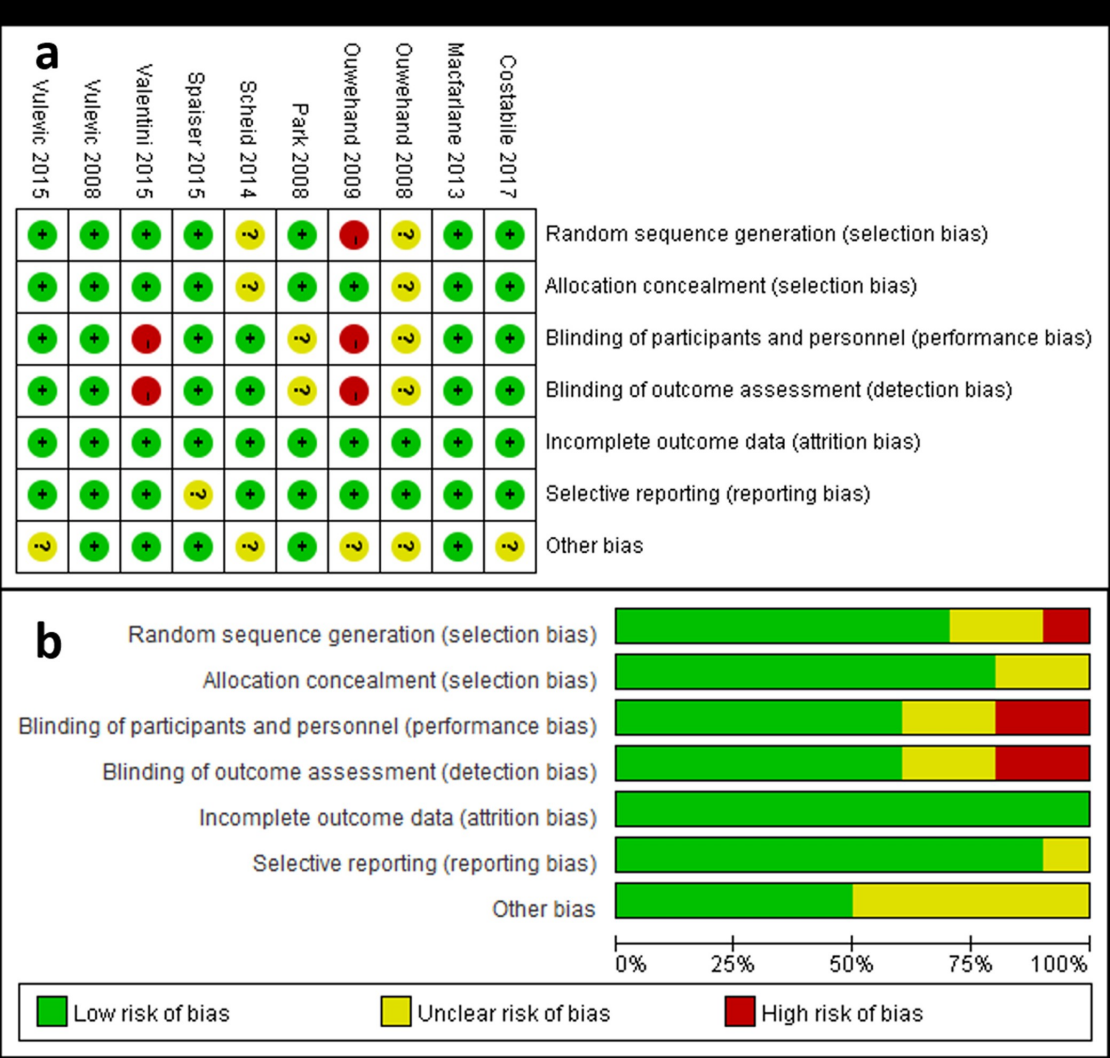
Risk of bias. a, Risk of bias summary: each risk of bias item for each included study; b, risk of bias graph: each risk of bias item presented as percentages across all included studies.

The quality assessment was performed to detect the potential risk biases. “Low risk”, “high risk” or “unclear risk” was categorized for all 10 included studies according to 7 sources of risk bias presented as sequence generation, allocation sequence concealment, blinding of individuals and personnel, blinding of outcome assessment, incomplete outcome data, selective outcome reporting and other potential sources of bias (Fig 2, Table 2). Taken together, no obvious attrition bias and reporting bias were observed, and the randomization and blinding in the included studies were considered adequate in the meta-analysis according to the Cochrane Handbook [17].

**Table 2:** Quality assessment of included study.

Study	Random sequence generation(selection bias)	Allocation concealment(selection bias)	Blinding of participants and presonel(preformance bias)	Blinding of outcome assessment(detection bias)	Incomplete outcome data(attrition bias)	Selective reporting (reporting bias)	Other bias
Park 2008	Low risk	Low risk	Unclear risk	Unclear risk	Low risk	Low risk	Low risk
Ouwehand 2008	Unclear risk	Unclear risk	Unclear risk	Unclear risk	Low risk	Low risk	Unclear risk
Vulevic 2008	Low risk	Low risk	Low risk	Low risk	Low risk	Low risk	Low risk
Ouwehand 2009	High risk	Low risk	High risk	High risk	Low risk	Low risk	Unclear risk
Macfarlane 2013	Low risk	Low risk	Low risk	Low risk	Low risk	Low risk	Low risk
Scheid 2014	Unclear risk	Unclear risk	Low risk	Low risk	Low risk	Low risk	Unclear risk
Valentini 2015	Low risk	Low risk	High risk	High risk	Low risk	Low risk	Low risk
Vulevic 2015	Low risk	Low risk	Low risk	Low risk	Low risk	Low risk	Unclear risk
Spaiser 2015	Low risk	Low risk	Low risk	Low risk	Low risk	Unclear risk	Low risk
Costabile 2017	Low risk	Low risk	Low risk	Low risk	Low risk	Low risk	Unclear risk

### Meta-analysis

**Fig 3:**
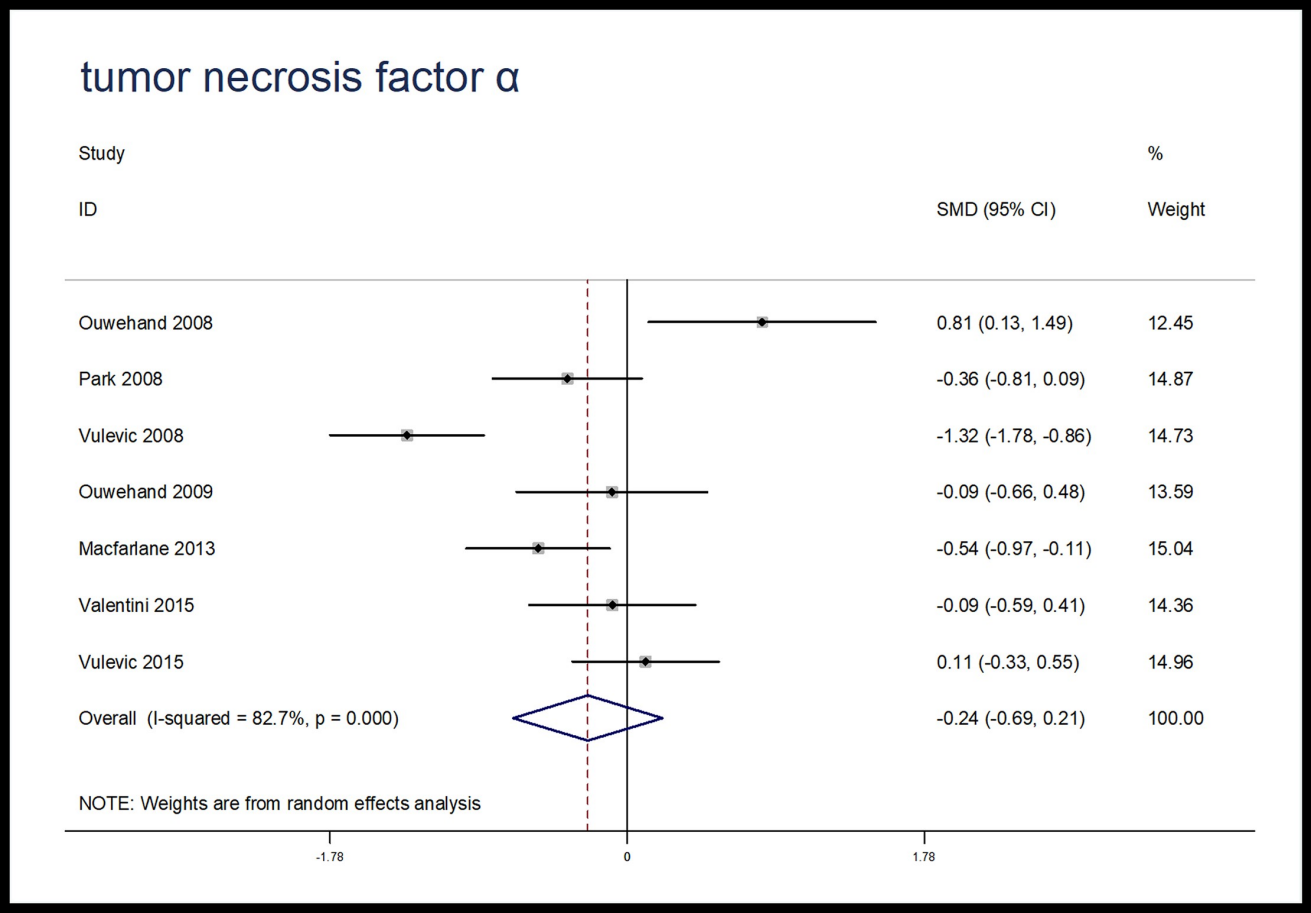
Forest plot for TNF-α, microbiota-driven therapy vs. placebo. TNF-α, tumor necrosis factor α; SMD, standard mean difference; CI, confidence interval. In the forest plot, solid vertical line represents for ineffective line, and the dashed red line represents for standard mean difference between microbiota-driven group and placebo group.

**Fig 4:**
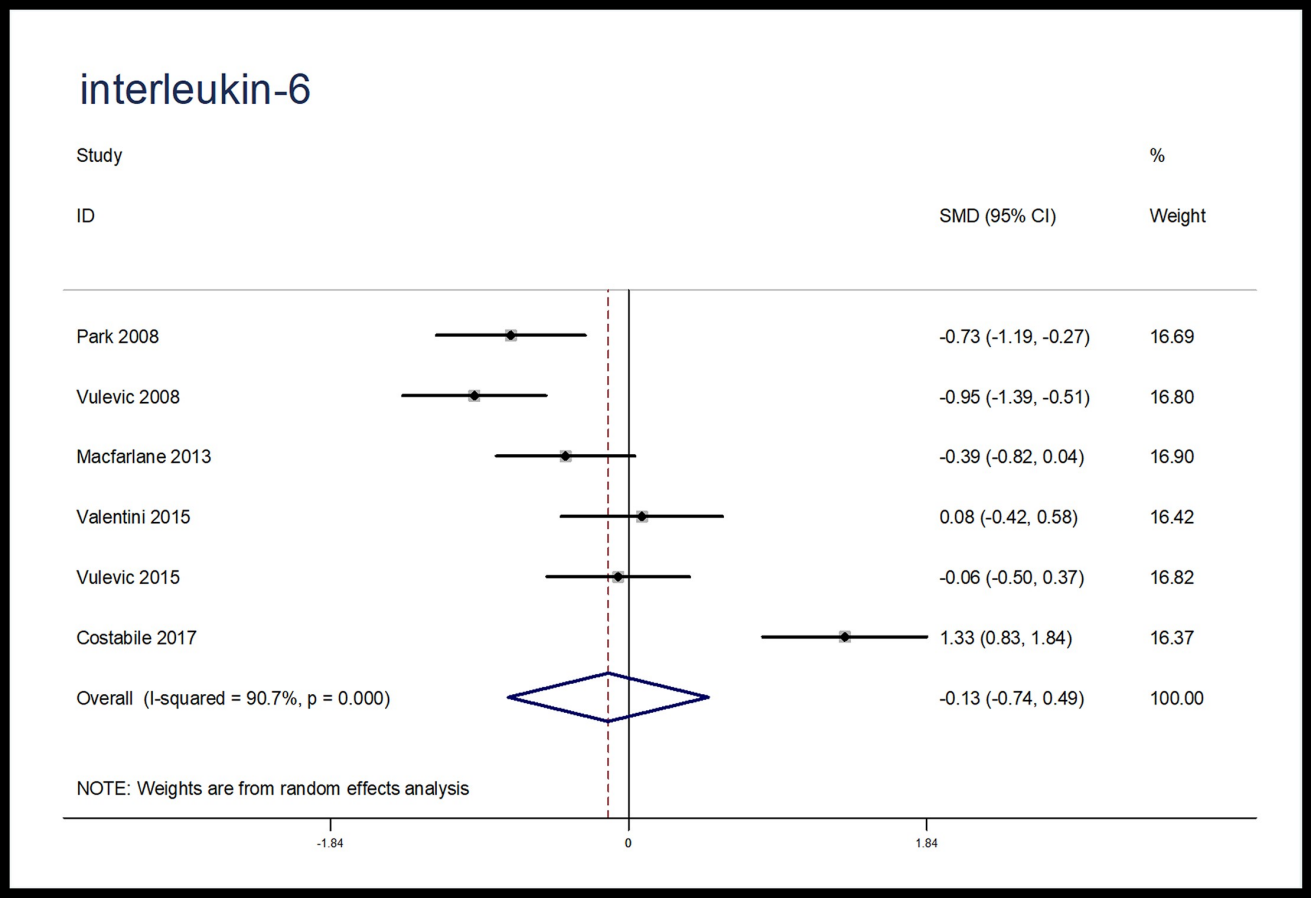
Forest plot for IL-6, microbiota-driven therapy vs. placebo. IL-6, interleukin-6; SMD, standard mean difference; CI, confidence interval. In the forest plot, solid vertical line represents for ineffective line, and the dashed red line represents for standard mean difference between microbiota-driven group and placebo group.

**Fig 5:**
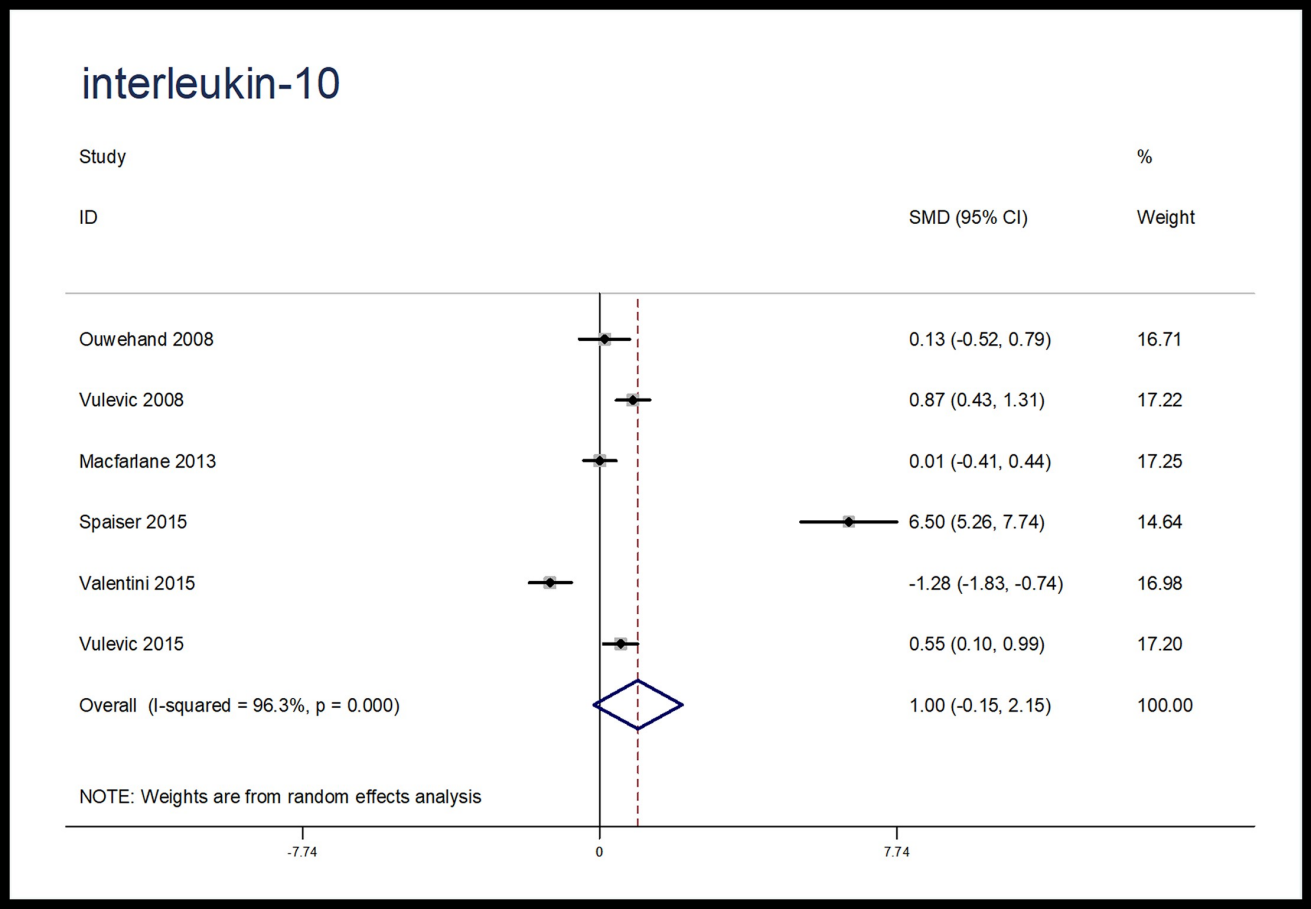
Forest plot for IL-10, microbiota-driven therapy vs. placebo. IL-10, interleukin-10; SMD, standard mean difference; CI, confidence interval. In the forest plot, solid vertical line represents for ineffective line, and the dashed red line represents for standard mean difference between microbiota-driven group and placebo group.

**Fig 6:**
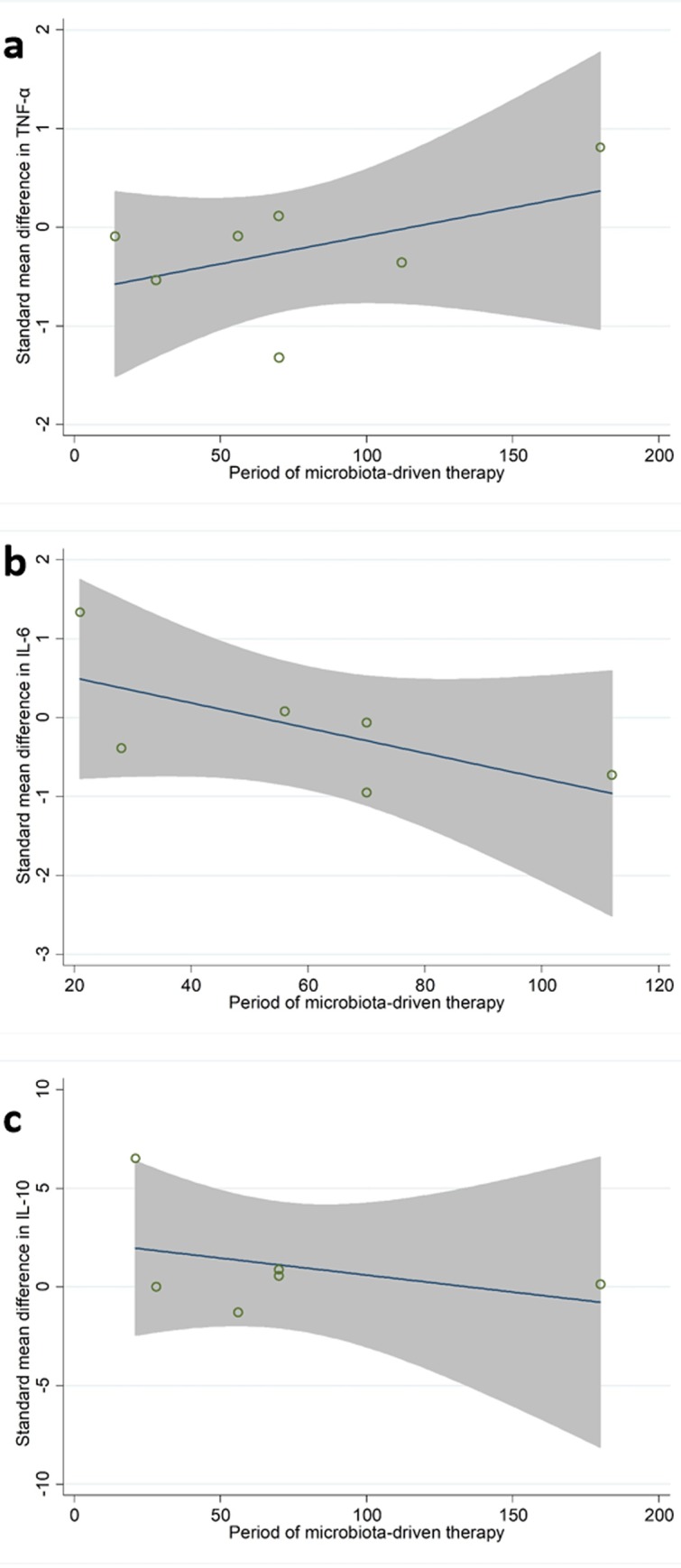
Meta-regression plot. a, standard mean difference in TNF-α according to period of microbiota-driven therapy (P = 0.28); b, standard mean difference in IL-6 according to period of microbiota-driven therapy (P = 0.16); c, standard mean difference in IL-10 according to period of microbiota-driven therapy (P = 0.28). TNF-α, tumor necrosis factor α; IL-6, interleukin-6; IL-10, interleukin-10.

When compared with placebo, microbiota-driven therapy did not decrease the levels of TNF-α (SMD, -0.24; 95% CI, -0.69 to 0.21; p = 0.30), IL-6 (SMD, -0.13; 95% CI, -0.74 to 0.49; p = 0.69) and IL-10 (SMD, 1.00; 95% CI, -0.15 to 2.15; p = 0.09) (Figs 3–5). There were significant heterogeneities among the studies regarding the outcomes of TNF-α, IL-6 and IL-10 (I^2^ = 82.7%, I^2^ = 90.7%, I^2^ = 96.3%), which were not obviously associated with the period of microbiota-driven therapy (P = 0.28, P = 0.16, P = 0.28, respectively, Fig 6).

**Fig 7:**
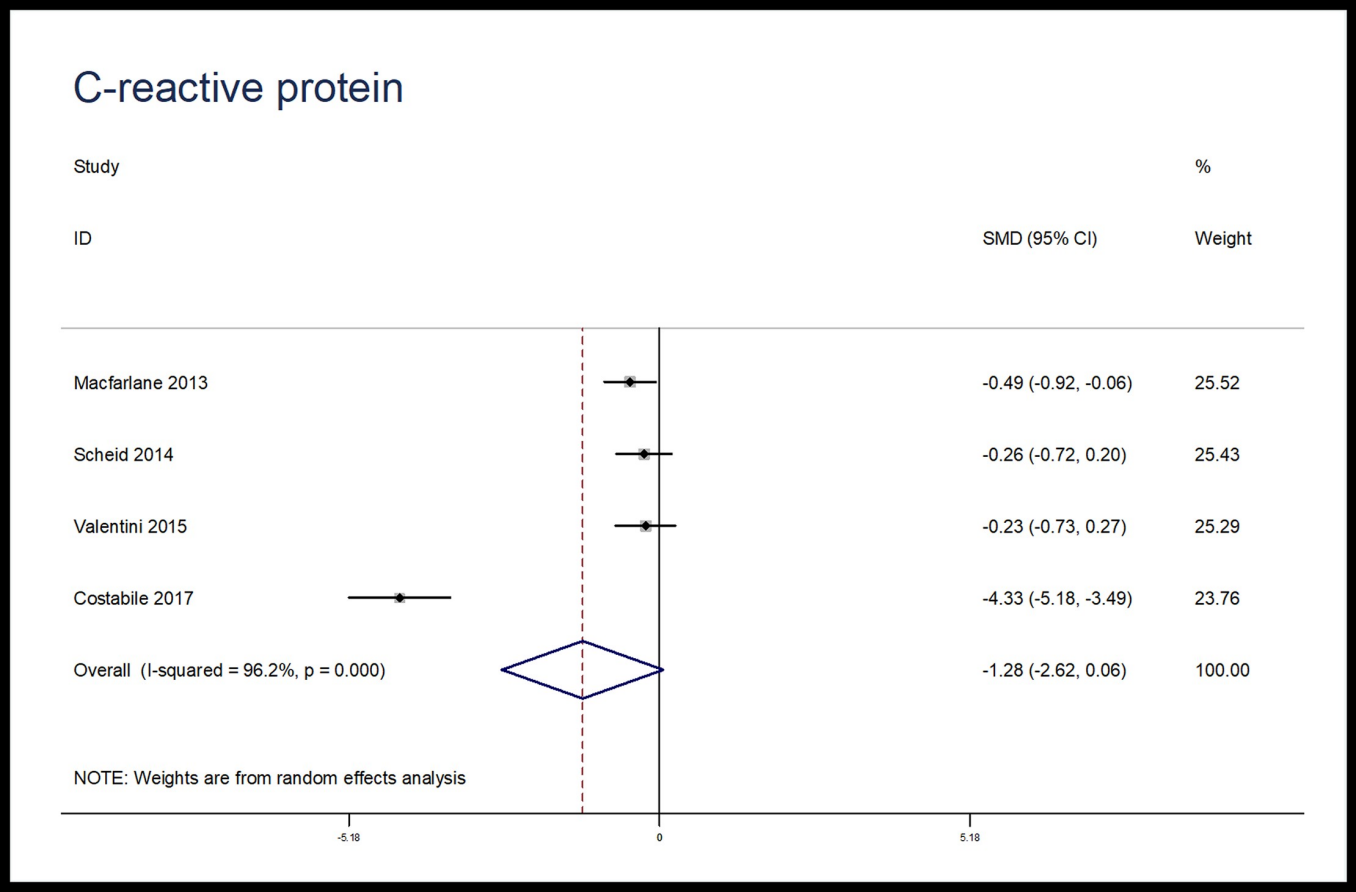
Forest plot for CRP, microbiota-driven therapy vs. placebo. CRP, C reactive protein; SMD, standard mean difference; CI, confidence interval. In the forest plot, solid vertical line represents for ineffective line, and the dashed red line represents for standard mean difference between microbiota-driven group and placebo group.

**Fig 8:**
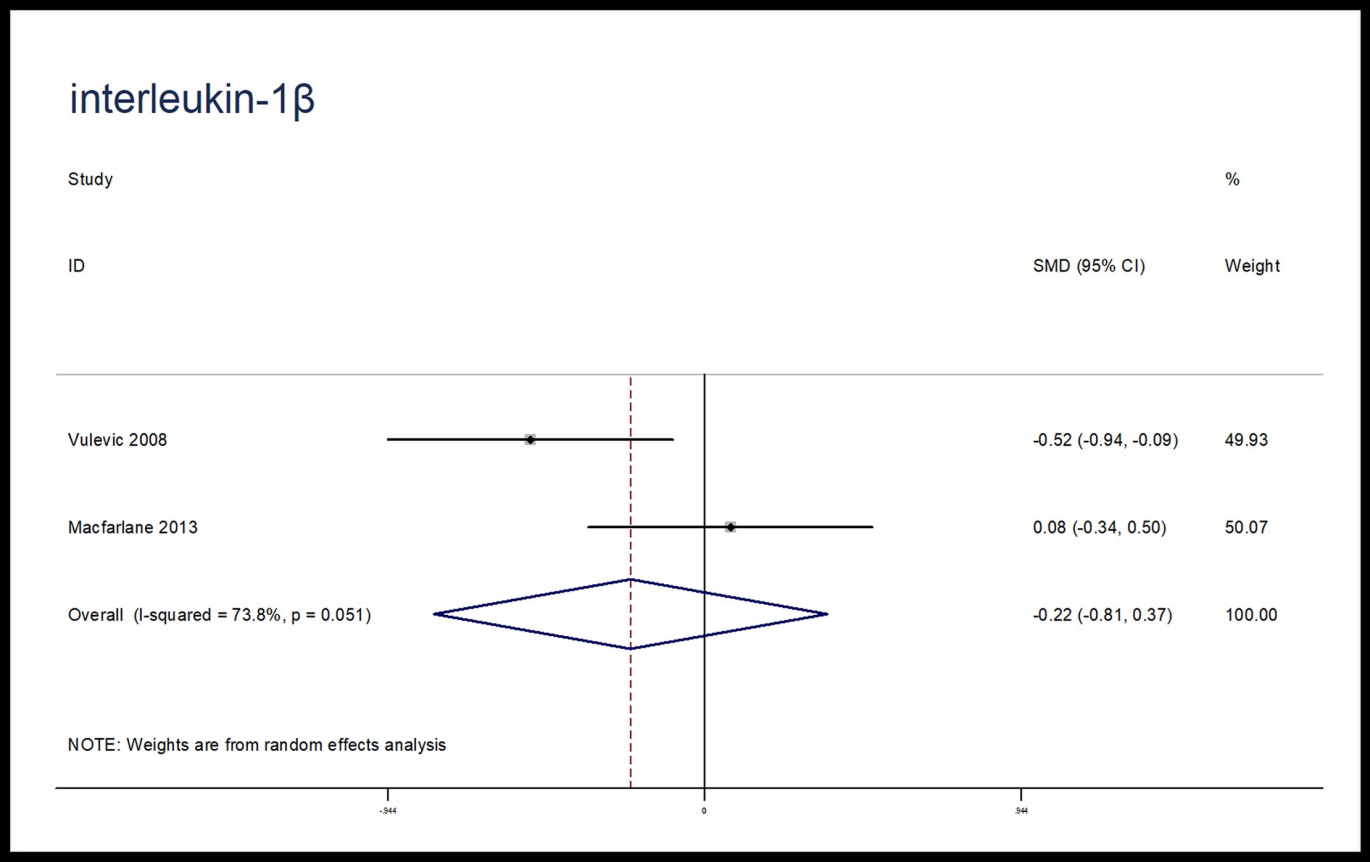
Forest plot for IL-8, microbiota-driven therapy vs. placebo. IL-8, interleukin-8; SMD, standard mean difference; CI, confidence interval. In the forest plot, solid vertical line represents for ineffective line, and the dashed red line represents for standard mean difference between microbiota-driven group and placebo group.

**Fig 9:**
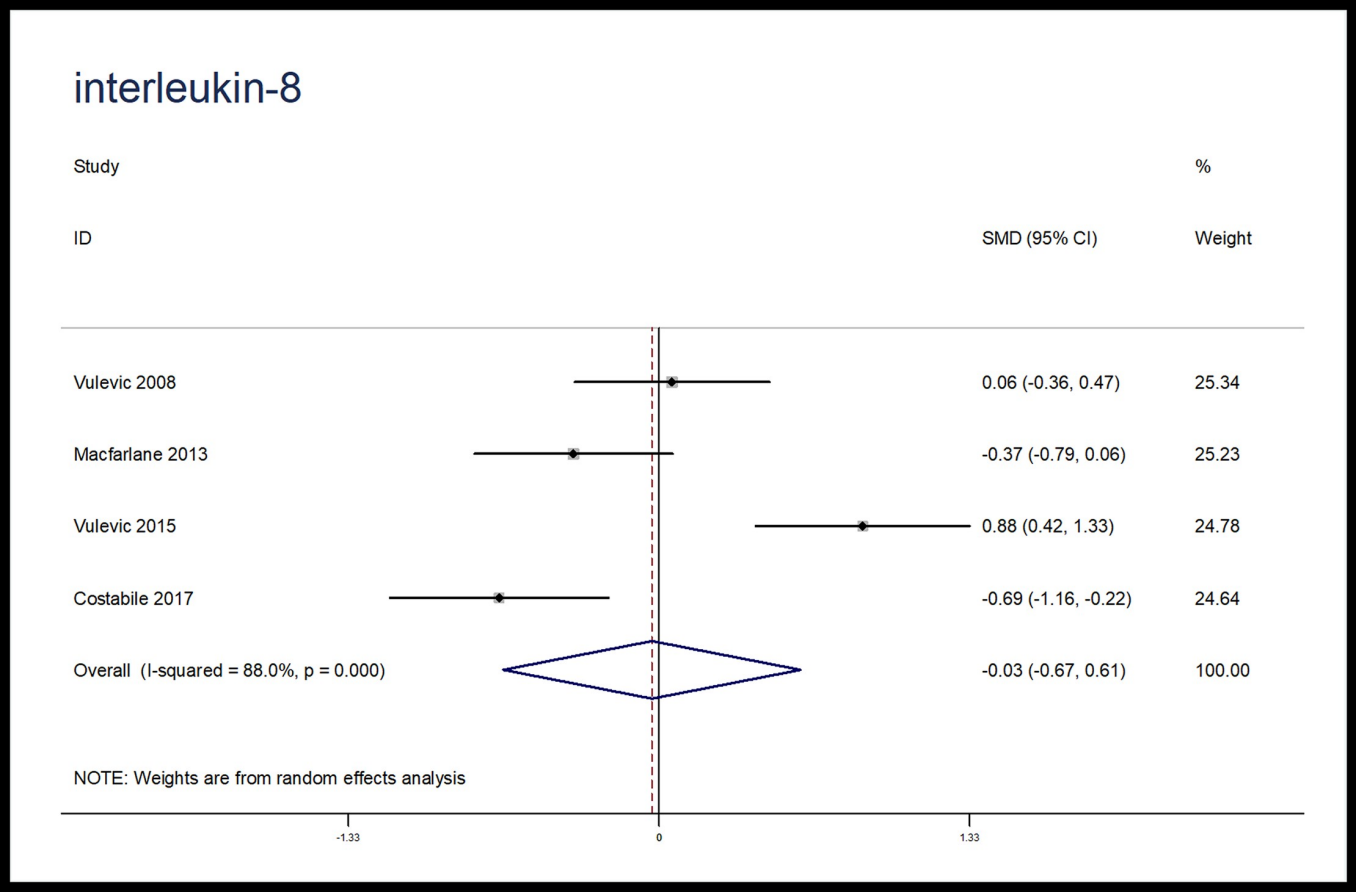
Forest plot for IL-1β, microbiota-driven therapy vs. placebo. IL-1β, interleukin-1β; SMD, standard mean difference; CI, confidence interval. In the forest plot, solid vertical line represents for ineffective line, and the dashed red line represents for standard mean difference between microbiota-driven group and placebo group.

**Fig 10:**
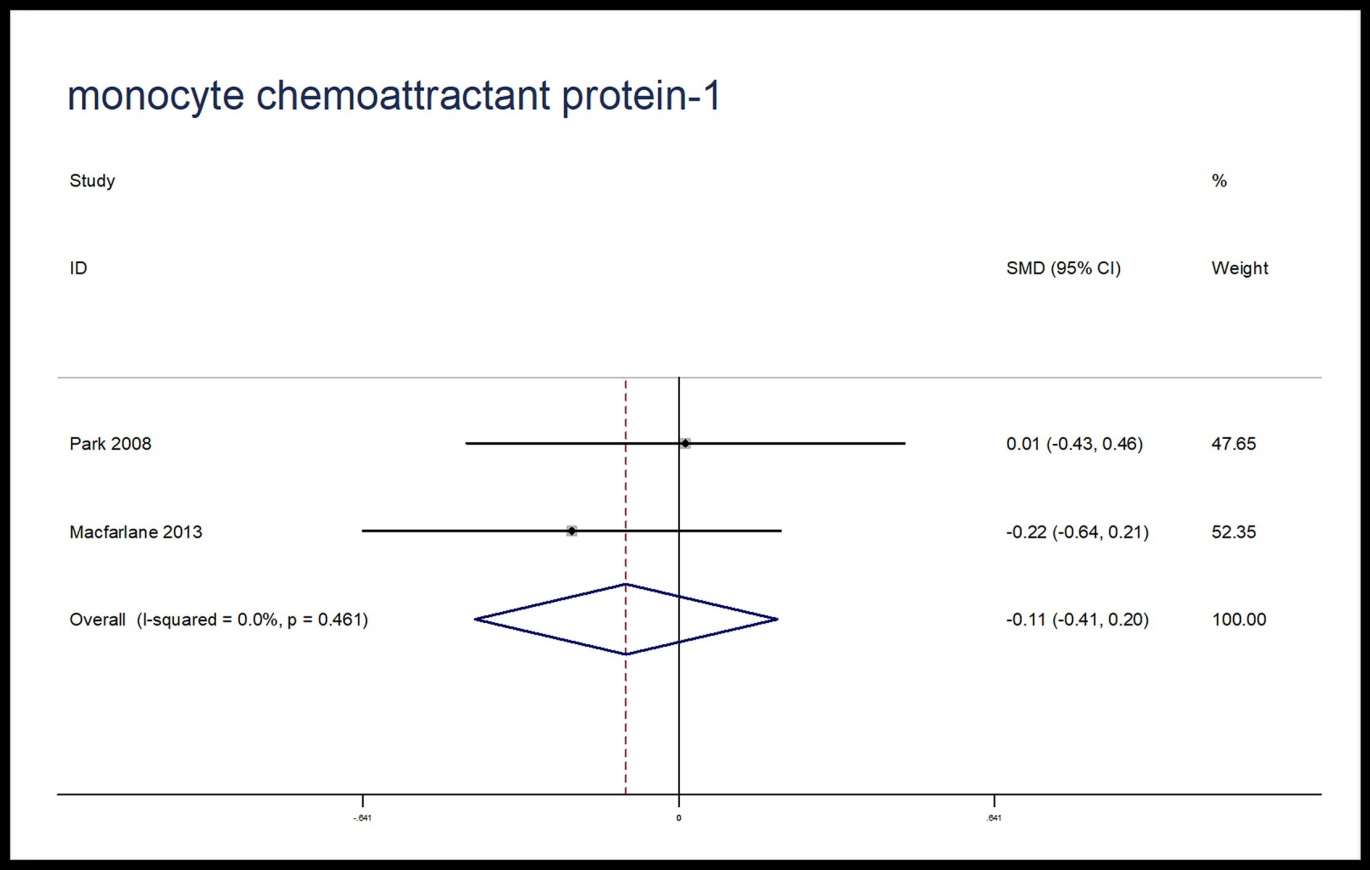
Forest plot for MCP-1, microbiota-driven therapy vs. placebo. MCP-1, monocyte chemoattractant protein-1; SMD, standard mean difference; CI, confidence interval. In the forest plot, solid vertical line represents for ineffective line, and the dashed red line represents for standard mean difference between microbiota-driven group and placebo group.

The effects of microbiota-driven therapy on other inflammatory markers, such as CRP, IL-1β, IL-8 and MCP-1 were also evaluated. Compared with placebo, microbiota-driven therapy did not decrease the levels of CRP (SMD, -1.28; 95% CI, -2.62 to 0.06; p = 0.06; I^2^ = 96.2%), IL-1β (SMD, -0.22; 95% CI, -0.81 to 0.37; p = 0.46; I^2^ = 73.8%), IL-8 (SMD, -0.03; 95% CI, -0.67 to 0.61; p = 0.93; I^2^ = 88.0%) and MCP-1 (SMD, -0.11; 95% CI, -0.41 to 0.20; p = 0.49; I^2^ = 0%). (Figs 7–10)

### Sensitivity analysis

To assure the reliability of the present meta-analysis, we performed sensitivity analysis to evaluate the robustness of the pooled results by eliminating each study at one time sequentially, indicating that the heterogeneity among the studies did not change significantly for the effect of microbiota-driven therapy on TNF-α, IL-6, and IL-10 (S3–S5 Tables).

### Publication bias

**Fig 11:**
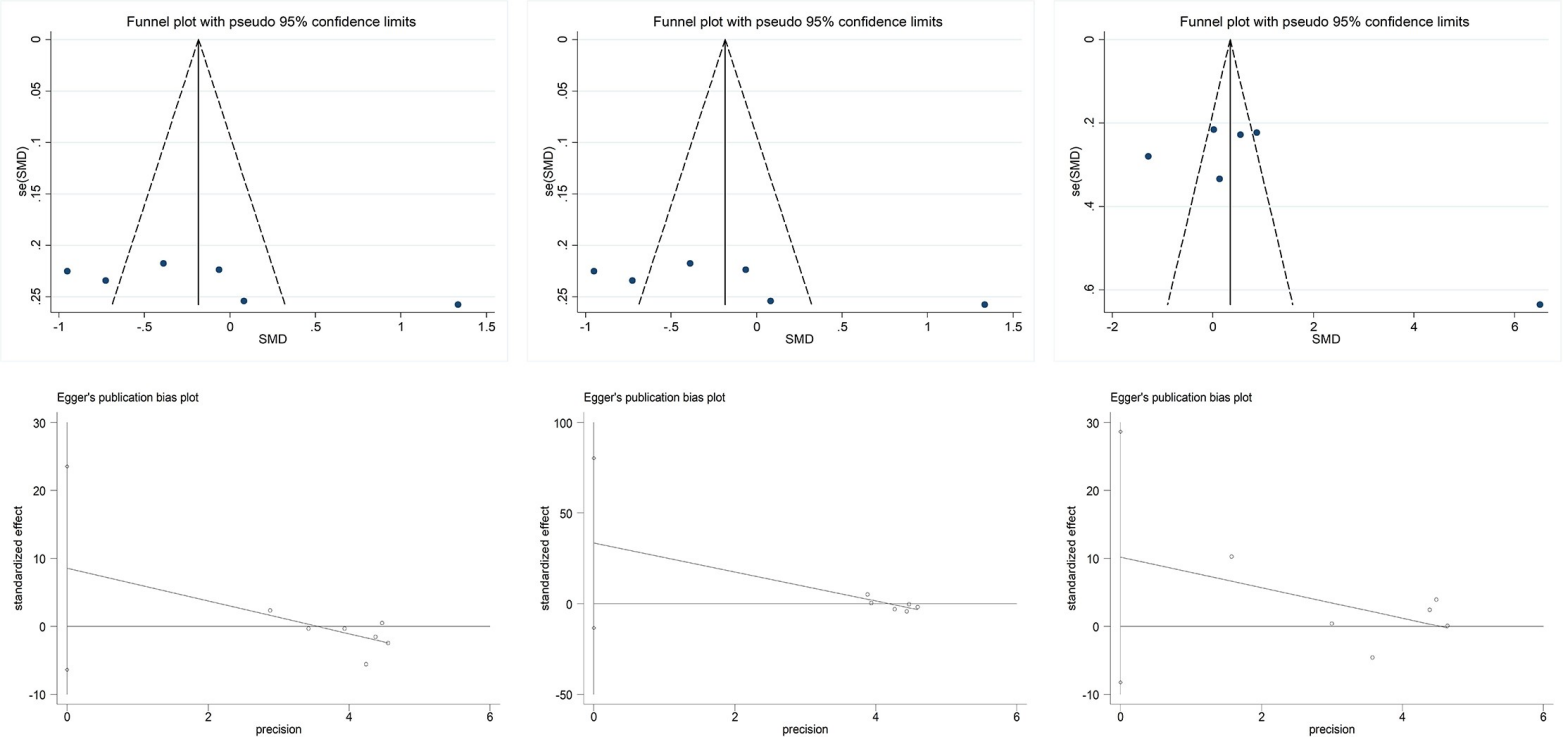
Publication bias. a, funnel plot for TNF-α; b, funnel plot for IL-6; c, funnel plot for IL-10; d, egger test for TNF-α(P = 0.37); e, egger test for IL-6(,P = 0.26); f, egger test for IL-10 (P = 0.20). TNF-α, tumor necrosis factor α; IL-6, interleukin-6; IL-10, interleukin-10; SMD, standard mean difference.

There was no significant publication bias identified in the analysis for the effect of microbiota-driven therapy on TNF-α, IL-6 and IL-10 using regression for funnel plot asymmetry (Egger’s test P = 0.37, P = 0.26, P = 0.20, respectively). The funnel plots created for the visual analysis of publication bias are presented in Fig 11.

## Discussion

To our knowledge, the present systematic review is the first to assess the effect of microbiota-driven therapy on inflammatory markers in elderly individuals and to provide a thorough synthesis of results from RCTs. After the treatment, no differences were observed between microbiota-driven therapy group and placebo group in the levels of TNF- α, CRP, IL-1 β, IL-6, IL-8, IL-10 and MCP-1.

Inflammatory markers (Il-1β, TNF-α, IL-6 and CRP) are continuously upregulated during the aging process[29], which were associated with reductions of muscle mass and sex hormones[30–32]. Accumulating evidence indicates that the low-grade chronic inflammatory state contributes to many age-related degenerative diseases that were previously not considered inflammatory disorders, including atherosclerosis[33–35], obesity[33,36], Alzheimer’s disease and Parkinson’s disease[34,37–40]. Recently, many studies have suggested the potential effect of microbiota-driven therapy on improving low-grade inflammatory states in some chronic diseases, such as type 2 diabetes[41–43] obesity[44,45] and inflammatory bowel disease (IBD)[46,47]. In addition, some studies also showed a benefit of microbiota-driven therapy in elderly individuals from the perspective of inflammation[12,13]. However, in most instances, the studies focusing on the effects of microbiota-driven therapy on inflammatory markers in elderly individuals had methodological limitations (mainly owing to small numbers of patients included), leaving effects of the therapy unproven.

In the present analysis, we provided the most reliable evidence to date, including 689 individuals from randomized trials to assess the specific effects of microbiota-driven therapy on inflammatory markers in elderly individuals. Contrary to findings from several smaller studies, we observed no significant effect of microbiota-driven therapy on inflammatory markers in elderly individuals. Notably, the effect sizes in the present study were independent of the period of microbiota-driven therapy. The sensitivity analyses of the present systematic review confirmed that the effect size was robust and was representative of all included studies. Therefore, this meta-analysis suggested that microbiota-driven therapy for decreasing the low-grade inflammation response in elderly individuals is not an effective option. However, we did not perform subgroup analysis based on the type of microbiota-driven therapy because of the limited number of included studies. Thus, it is necessary to perform an analysis to compare the differences in decreasing inflammatory markers among prebiotic, probiotic and symbiotic when there are enough available studies. In addition, the dosage of microbiota-driven therapy could also influence the present results, the meta-regression based on dosage of microbiota-driven therapy is also needed in the future. Several limitations should be illustrated in the present meta-analysis. First, the included studies were heterogeneous because of population characteristics and the period of microbiota-driven therapy. However, meta-regression and sensitivity analyses were performed to assure the reliability of the present meta-analysis. Second, there were a limited number of eligible RCTs, and most of them included relatively small populations; thus, the impact of the variables (e.g., sex, region, etc.) on the outcomes could not be evaluated. The analyses based on these variables should be performed when there are enough data in the future.

## Conclusion

This meta-analysis of available RCTs does not suggest any significant benefit of microbiota-driven therapy in improving the low-grade chronic inflammatory state of elderly individuals.

## Supporting information

S1 Table PRISMA checklist. (DOCX)

S2 Table Searching strategies. (DOCX)

S3 Table Sensitivity analysis on TNF-α. (DOCX)

S4 Table Sensitivity analysis on IL-6. (DOCX)

S5 Table Sensitivity analysis on IL-10. (DOCX)
