# Supplementary material for: Effects of microbiota-driven therapy on inflammatory responses in elderly individuals: A systematic review and meta-analysis
Source: PLoS One. 2019 Feb 6;14(2):e0211233. doi: 10.1371/journal.pone.0211233 (PMC6364922; doi:10.1371/journal.pone.0211233)
Supplement: S2 Table — (DOCX) [file pone.0211233.s002.docx]

S2 Table. Searching strategies

| Database | Search | Search Strings | Hits(2018.11.11) |
| --- | --- | --- | --- |
| PubMed | #1 | aged OR elderly[All Fields] | 4987946 |
|  | #2 | (((((((((VSL) OR bifidobacteria) OR lactococcus) OR enterococcus) OR saccharomyces) OR lactobacilli) OR streptococcus) OR synbiotic) OR probiotic) OR prebiotic[All Fields] | 287268 |
|  | #3 | C Reactive Protein OR Protein, C-Reactive OR CRP[All Fields] | 80350 |
|  | #4 | Tumor Necrosis Factor alpha OR Cachectin OR Tumor Necrosis Factor Ligand Superfamily Member 2 OR Tumor Necrosis Factor OR TNF Superfamily, Member 2 OR TNFalpha OR TNF-alpha OR TNF-α[All Fields] | 240323 |
|  | #5 | (((((((((((((((((((((((((((((Interleukin 6) OR IL6) OR B-Cell Stimulatory Factor 2) OR B-Cell Stimulatory Factor-2) OR Differentiation Factor-2, B-Cell) OR Differentiation Factor 2, B Cell) OR B-Cell Differentiation Factor-2) OR B Cell Differentiation Factor 2) OR BSF-2) OR Hybridoma Growth Factor) OR Growth Factor, Hybridoma) OR IFN-beta 2) OR Plasmacytoma Growth Factor) OR Growth Factor, Plasmacytoma) OR Hepatocyte-Stimulating Factor) OR Hepatocyte Stimulating Factor) OR MGI-2) OR Myeloid Differentiation-Inducing Protein) OR Differentiation-Inducing Protein, Myeloid) OR Myeloid Differentiation Inducing Protein) OR B-Cell Differentiation Factor) OR B Cell Differentiation Factor) OR Differentiation Factor, B-Cell) OR Differentiation Factor, B Cell) OR IL-6) OR Interferon beta-2) OR Interferon beta 2) OR beta-2, Interferon) OR B Cell Stimulatory Factor-2) OR B Cell Stimulatory Factor 2[All Fields] | 115794 |
|  | #6 | ((((((((((((((((((((((((((((((((Interleukin 8) OR IL8) OR Monocyte-Derived Neutrophil Chemotactic Factor) OR Neutrophil Activation Factor) OR Neutrophil-Activating Peptide, Lymphocyte-Derived) OR Lymphocyte-Derived Neutrophil-Activating Peptide) OR Neutrophil Activating Peptide, Lymphocyte Derived) OR Neutrophil-Activating Peptide, Monocyte-Derived) OR Monocyte-Derived Neutrophil-Activating Peptide) OR Neutrophil Activating Peptide, Monocyte Derived) OR Alveolar Macrophage Chemotactic Factor-I) OR Alveolar Macrophage Chemotactic Factor I) OR AMCF-I) OR Anionic Neutrophil-Activating Peptide) OR Anionic Neutrophil Activating Peptide) OR Neutrophil-Activating Peptide, Anionic) OR Peptide, Anionic Neutrophil-Activating) OR Chemokine CXCL8) OR CXCL8, Chemokine) OR Chemokines, CXCL8) OR CXCL8 Chemokines) OR Chemotactic Factor, Macrophage-Derived) OR Chemotactic Factor, Macrophage Derived) OR Macrophage-Derived Chemotactic Factor) OR Chemotactic Factor, Neutrophil) OR Neutrophil Chemotactic Factor) OR Chemotactic Factor, Neutrophil, Monocyte-Derived) OR CXCL8 Chemokine) OR Chemokine, CXCL8) OR Granulocyte Chemotactic Peptide-Interleukin-8) OR Chemotactic Peptide-Interleukin-8, Granulocyte) OR Granulocyte Chemotactic Peptide Interleukin 8) OR IL-8[All Fields] | 44921 |
|  | #7 | ((((Interleukin 10) OR IL10) OR IL-10) OR CSIF-10) OR Cytokine Synthesis Inhibitory Factor[All Fields] | 56079 |
|  | #8 | ((((((interleukin-1β) OR Interleukin 1beta) OR IL-1 beta) OR Interleukin-1 beta) OR Interleukin 1 beta) OR Catabolin) OR IL-1β[All Fields] | 65191 |
|  | #9 | (((((((((((((CCL2) OR (Monocyte Chemotactic and Activating Factor)) OR Monocyte Chemoattractant Protein-1) OR Chemoattractant Protein-1, Monocyte) OR Monocyte Chemoattractant Protein 1) OR Chemokines CCL2) OR CCL2, Chemokines) OR CCL2 Chemokine) OR Chemokine, CCL2) OR Chemokine (C-C Motif) Ligand 2) OR Monocyte Chemotactic Protein-1) OR Monocyte Chemotactic Protein-1) OR Monocyte Chemotactic Protein 1) OR MCP-1[All Fields] | 13831 |
|  | #10 | #3 OR #4 OR #5 OR #6 OR #7 OR #8 OR #9 | 442378 |
|  | #11 | #1 and #2 and #10 | 775 |
| EMBASE | #1 | 'aged'/exp OR aged | 4067238 |
|  | #2 | 'elderly'/exp OR elderly | 2,891784 |
|  | #3 | #1 OR #2 | 4139426 |
|  | #4 | 'probiotic'/exp OR probiotic | 32723 |
|  | #5 | 'prebiotic'/exp OR prebiotic | 8927 |
|  | #6 | 'synbiotic'/exp OR synbiotic | 1558 |
|  | #7 | 'streptococcus'/exp OR streptococcus | 158621 |
|  | #8 | 'lactobacilli'/exp OR lactobacilli | 43238 |
|  | #9 | 'saccharomyces'/exp OR saccharomyces | 113010 |
|  | #10 | 'enterococcus'/exp OR enterococcus | 48889 |
|  | #11 | 'lactococcus'/exp OR lactococcus | 8564 |
|  | #12 | vsl | 1276 |
|  | #13 | bifidobacteria | 4550 |
|  | #14 | #4 OR #5 OR #6 OR #7 OR #8 OR #9 OR #10 OR #11 OR #12 OR #13 | 367235 |
|  | #15 | 'c reactive protein'/exp OR 'c reactive protein' | 157180 |
|  | #16 | 'interleukin 6'/exp OR 'interleukin 6' | 215748 |
|  | #17 | 'interleukin 8'/exp OR 'interleukin 8' | 72243 |
|  | #18 | 'interleukin 10'/exp OR 'interleukin 10' | 103729 |
|  | #19 | 'interleukin 1beta'/exp OR 'interleukin 1beta' | 110599 |
|  | #20 | 'monocyte chemotactic protein 1'/exp OR 'monocyte chemotactic protein 1' | 42791 |
|  | #21 | #15 OR #16 OR #17 OR #18 OR #19 OR #20 | 511639 |
|  | #22 | 'controlled study' | 6302104 |
|  | #23 | #21 AND #22 | 655 |
| Cochrane Library | #1 | MeSH descriptor: [Probiotics] explode all trees | 1688 |
|  | #2 | MeSH descriptor: [Prebiotics] explode all trees | 218 |
|  | #3 | MeSH descriptor: [Synbiotics] explode all trees | 103 |
|  | #4 | (streptococcus):ti,ab,kw | 3776 |
|  | #5 | (lactobacilli):ti,ab,kw | 762 |
|  | #6 | (saccharomyces):ti,ab,kw | 361 |
|  | #7 | (enterococcus):ti,ab,kw | 801 |
|  | #8 | (lactococcus):ti,ab,kw | 71 |
|  | #9 | (bifidobacteria):ti,ab,kw | 572 |
|  | #10 | (VSL):ti,ab,kw | 139 |
|  | #11 | (probiotic):ti,ab,kw | 4301 |
|  | #12 | (prebiotic):ti,ab,kw | 950 |
|  | #13 | (synbiotic):ti,ab,kw | 379 |
|  | #14 | #1 or #2 or #3 or #4 or #5 or #6 or #7 or #8 or #9 or #10 or #11 or #12 or #13 | 9544 |
|  | #15 | (aged):ti,ab,kw | 528300 |
|  | #16 | (elderly):ti,ab,kw | 28829 |
|  | #17 | #15 or #16 | 533747 |
|  | #18 | MeSH descriptor: [C-Reactive Protein] explode all trees | 4236 |
|  | #19 | (C reactive protein):ti,ab,kw (Word variations have been searched) | 13361 |
|  | #20 | MeSH descriptor: [Interleukin-1beta] explode all trees | 375 |
|  | #21 | (interleukin 1beta):ti,ab,kw | 2044 |
|  | #22 | MeSH descriptor: [Tumor Necrosis Factor-alpha] explode all trees | 2888 |
|  | #23 | (Tumor Necrosis Factor alpha):ti,ab,kw | 7011 |
|  | #24 | MeSH descriptor: [Interleukin-6] explode all trees | 2819 |
|  | #25 | (Interleukin 6):ti,ab,kw | 12219 |
|  | #26 | MeSH descriptor: [Interleukin-8] explode all trees | 764 |
|  | #27 | (Interleukin 8):ti,ab,kw | 7341 |
|  | #28 | MeSH descriptor: [Interleukin-10] explode all trees | 748 |
|  | #29 | (Interleukin 10):ti,ab,kw | 7163 |
|  | #30 | MeSH descriptor: [Chemokine CCL2] explode all trees | 252 |
|  | #31 | (monocyte chemotactic protein 1):ti,ab,kw | 697 |
|  | #32 | #18 or #19 or #20 or #21 or #22 or #23 or #24 or #25 or #26 or #27 or #28 or #29 or #30 or #31 | 27387 |
|  | #33 | #14 and #17 and #32 | 349 |
