# Supplementary material for: Effects of microbiota-driven therapy on inflammatory responses in elderly individuals: A systematic review and meta-analysis
Source: PLoS One. 2019 Feb 6;14(2):e0211233. doi: 10.1371/journal.pone.0211233 (PMC6364922; doi:10.1371/journal.pone.0211233)
Supplement: S4 Table — (DOCX) [file pone.0211233.s004.docx]

S4 Table. Sensitivity analysis on IL-6

| study | effect size | low CI | up CI | I^2^ | P_h_ |
| --- | --- | --- | --- | --- | --- |
| Costabile 2017 | -0.42 | -0.79 | -0.04 | 70.90% | 0.008 |
| Macfarlane 2013 | -0.07 | -0.83 | 0.69 | 92.40% | <0.001 |
| Park 2008 | -0.005 | -0.71 | 0.7 | 91.60% | <0.001 |
| Valentini 2015 | -0.17 | -0.9 | 0.57 | 92.40% | <0.001 |
| Vulevic 2008 | 0.04 | -0.61 | 0.69 | 89.90% | <0.001 |
| Vulevic 2015 | -0.14 | -0.9 | 0.62 | 92.50% | <0.001 |

Abbreviations: IL-6, interleukin-6；CI, confidence interval.
