# Supplementary material for: Effects of microbiota-driven therapy on inflammatory responses in elderly individuals: A systematic review and meta-analysis
Source: PLoS One. 2019 Feb 6;14(2):e0211233. doi: 10.1371/journal.pone.0211233 (PMC6364922; doi:10.1371/journal.pone.0211233)
Supplement: S5 Table — (DOCX) [file pone.0211233.s005.docx]

S5 Table. Sensitivity analysis on IL-10

| study | effect size | low CI | up CI | I^2^ | P_h_ |
| --- | --- | --- | --- | --- | --- |
| Macfarlane 2013 | 1.25 | -0.24 | 2.73 | 97.00% | <0.001 |
| Ouwehand 2008 | 1.2 | -0.17 | 2.56 | 97.10% | <0.001 |
| Spaiser 2015 | 0.07 | -0.63 | 0.76 | 90.00% | <0.001 |
| Valentini 2015 | 1.45 | 0.26 | 2.65 | 95.90% | <0.001 |
| Vulevic 2008 | 1.06 | -0.39 | 2.51 | 96.90% | <0.001 |
| Vulevic 2015 | 1.13 | -0.34 | 2.61 | 97.10% | <0.001 |

Abbreviations: IL-10, interleukin-10；CI, confidence interval.
